# (2*R*,4*R*)-3-(*tert*-But­oxy­carbon­yl)-2-(3-chloro­phen­yl)-1,3-thia­zolidine-4-carb­oxy­lic acid monohydrate

**DOI:** 10.1107/S1600536810042133

**Published:** 2010-10-23

**Authors:** Zhong-Cheng Song, Ying Guo, Wen-Hong Liu, Li-Chun Hu, Sheng-Nan Cai

**Affiliations:** a Bioengineering Department, Zhejiang Traditional Chinese Medicine University, Hangzhou 310053, People’s Republic of China

## Abstract

In the title compound, C_15_H_18_ClNO_4_S·H_2_O, the thia­zolidine ring displays a half-chair conformation. In the crystal, the water mol­ecules are linked to the organic acid mol­ecules *via* inter­molecular O—H⋯O hydrogen bonds.

## Related literature

For applications of thia­zolidine derivatives, see: Kallen (1971 ▶); Seki *et al.* (2004 ▶); Song *et al.* (2009 ▶).
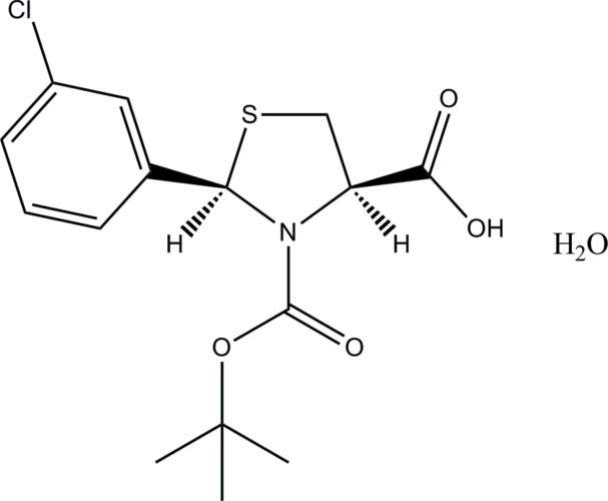


## Experimental

### 

#### Crystal data

C_15_H_18_ClNO_4_S·H_2_O
*M* *_r_* = 361.83
Monoclinic, 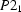

*a* = 8.2460 (16) Å
*b* = 5.9660 (12) Å
*c* = 18.132 (4) Å
β = 99.81 (3)°
*V* = 879.0 (3) Å^3^
*Z* = 2
Mo *K*α radiation
μ = 0.36 mm^−1^
*T* = 293 K
0.20 × 0.17 × 0.15 mm

#### Data collection

Enraf–Nonius CAD-4 diffractometer
Absorption correction: ψ scan (*ABSCOR*; Higashi, 1995 ▶) *T*_min_ = 0.932, *T*_max_ = 0.948
3417 measured reflections
3182 independent reflections
2169 reflections with *I* > 2σ(*I*)
*R*_int_ = 0.080
3 standard reflections every 200 reflections  intensity decay: 1%

#### Refinement

*R*[*F* ^2^ > 2σ(*F* ^2^)] = 0.068
*wR*(*F* ^2^) = 0.167
*S* = 1.03
3182 reflections
208 parameters
1 restraint
H-atom parameters constrained
Δρ_max_ = 0.19 e Å^−3^
Δρ_min_ = −0.28 e Å^−3^
Absolute structure: Flack (1983 ▶), 1451 Friedel pairs
Flack parameter: −0.11 (16)

### 

Data collection: *CAD-4 EXPRESS* (Enraf–Nonius, 1994 ▶); cell refinement: *CAD-4 EXPRESS*; data reduction: *XCAD4* (Harms & Wocadlo, 1995 ▶); program(s) used to solve structure: *SHELXTL* (Sheldrick, 2008 ▶); program(s) used to refine structure: *SHELXTL*; molecular graphics: *SHELXTL*; software used to prepare material for publication: *SHELXTL*.

## Supplementary Material

Crystal structure: contains datablocks global, I. DOI: 10.1107/S1600536810042133/xu5054sup1.cif

Structure factors: contains datablocks I. DOI: 10.1107/S1600536810042133/xu5054Isup2.hkl

Additional supplementary materials:  crystallographic information; 3D view; checkCIF report

## Figures and Tables

**Table 1:** Hydrogen-bond geometry (Å, °)

*D*—H⋯*A*	*D*—H	H⋯*A*	*D*⋯*A*	*D*—H⋯*A*
O2—H2*C*⋯O5^i^	0.82	1.80	2.620 (6)	177
O5—H5*A*⋯O3^ii^	0.85	2.20	2.890 (5)	139
O5—H5*B*⋯O3^iii^	0.85	2.37	2.827 (5)	114

Symmetry codes: (i) 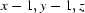; (ii) 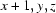; (iii) 
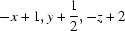
.

**Fig. 1.**
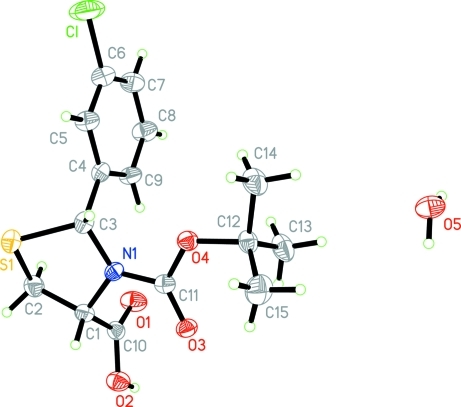
The molecular structure of the title compounds with atom labels and the 30% probability displacement ellipsoids for H atoms.

**Fig. 2.**
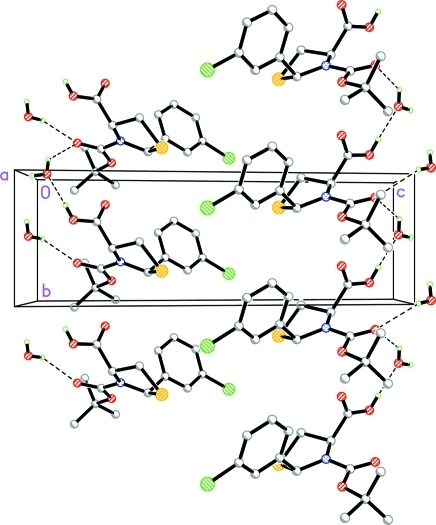
Molecular packing of the title compound, viewed along the a axis. Hydrogen bonds are shown as dashed lines.

## supplementary crystallographic information

### Comment

The steric course of the reaction between *L*-cysteine and aldehydes deserved much attention because this reaction had been implicated in several biochemical processes (Kallen, 1971). An analogous condensation reaction constituted the first step in the syntheses of important natural products, such as penicillin and biotin (Seki *et al.*, 2004). Therefore, thiazolidine derivatives have become especially noteworthy in recent years (Song *et al.* 2009). In the present work, the structure of the title new compound is reported.

The compound consists of a (2*R*,4*R*)-3-(*tert*-butoxycarbonyl)-2-(3-chlorophenyl)thiazolidine-4-carboxylic acid molecule and a water molecule of crystallization (Fig. 1). The torsion angles C12—O4—C11—O3, C12—O4—C11—N1, and N1—C1—C2—S1 are 3.2 (5), 5.5 (5), and 37.1 (5)°, respectively. The S1 atom is located 0.762 (6)Å from the least-squares plane defined by C2/C1/N1/C3. In the crystal structure, the (2*R*,4*R*)-3-(*tert*-butoxycarbonyl)-2- (3-chlorophenyl)thiazolidine-4-carboxylic acid molecules are linked by water molecules through intermolecular O–H···O hydrogen bonds (Table 1), forming chains running along the *b* axis (Fig. 2).

### Experimental

Cysteine (0.121 g, 1.0 mmol) and appropriate aldehyde (1.0 mmol) in ethanol (25 ml) was stirred at room temperature for 8 h, and the solid separated was collected, washed with diethyl ether and dried to obtain (2RS,4*R*)-2-(3-chlorophenyl)thiazolidine-4-carboxylic acid (TCA). A mixture of TCA (1.0 mmol) and appropriate NaOH (10%, 1.0 mmol) in dioxane (25 ml) was stirred at ice-water temperature for 2 h. BOC_2_O (1.0 mmol) was added and stirred at ice-water temperature for 1 h and then at room temperature for 5 h. Most of the solvent was extracted and appropriate amount of water was added to adjust to neutral pH value. Ethyl acetate was added and extracted (50 ml), and washed with appropriate saturated aqueous solution of common salt, and dried with anhydrous magnesium sulfate. Solvent was extracted to dry to obtain whiter solids (2*R*,4*R*)-3-(*tert*-butoxycarbonyl)-2-(3-chlorophenyl)thiazolidine-4-carboxylic acid hydrate

### Refinement

H atoms were positioned geometrically and refined using the riding-model approximation, with C–H = 0.93–0.97 Å, O–H = 0.82–0.85 Å, and *U*_iso_(H) = 1.2*U*_eq_(C,O) or *U*_iso_(H) = 1.5*U*_eq_(methyl C).

### Crystal data

**Table:**

C_15_H_18_ClNO_4_S·H_2_O	*F*(000) = 380
*M**_r_* = 361.83	*D*_x_ = 1.367 Mg m^−^^3^
Monoclinic, *P*2_1_	Mo *K*α radiation, λ = 0.71073 Å
Hall symbol: P 2yb	Cell parameters from 25 reflections
*a* = 8.2460 (16) Å	θ = 9–12°
*b* = 5.9660 (12) Å	µ = 0.36 mm^−^^1^
*c* = 18.132 (4) Å	*T* = 293 K
β = 99.81 (3)°	Block, colorless
*V* = 879.0 (3) Å^3^	0.20 × 0.17 × 0.15 mm
*Z* = 2	

### Data collection

**Table:**

Enraf–Nonius CAD-4 diffractometer	2169 reflections with *I* > 2σ(*I*)
Radiation source: fine-focus sealed tube	*R*_int_ = 0.080
graphite	θ_max_ = 25.3°, θ_min_ = 1.1°
ω/2θ scans	*h* = 0→9
Absorption correction: ψ scan (*ABSCOR*; Higashi, 1995)	*k* = −7→7
*T*_min_ = 0.932, *T*_max_ = 0.948	*l* = −21→21
3417 measured reflections	3 standard reflections every 200 reflections
3182 independent reflections	intensity decay: 1%

### Refinement

**Table:**

Refinement on *F*^2^	Secondary atom site location: difference Fourier map
Least-squares matrix: full	Hydrogen site location: inferred from neighbouring sites
*R*[*F*^2^ > 2σ(*F*^2^)] = 0.068	H-atom parameters constrained
*wR*(*F*^2^) = 0.167	*w* = 1/[σ^2^(*F*_o_^2^) + (0.0664*P*)^2^ + 0.2379*P*] where *P* = (*F*_o_^2^ + 2*F*_c_^2^)/3
*S* = 1.03	(Δ/σ)_max_ = 0.001
3182 reflections	Δρ_max_ = 0.19 e Å^−^^3^
208 parameters	Δρ_min_ = −0.28 e Å^−^^3^
1 restraint	Absolute structure: Flack (1983), 1451 Friedel pairs
Primary atom site location: structure-invariant direct methods	Flack parameter: −0.11 (16)

### Special details

**Table:**

Geometry. All e.s.d.'s (except the e.s.d. in the dihedral angle between two l.s. planes) are estimated using the full covariance matrix. The cell e.s.d.'s are taken into account individually in the estimation of e.s.d.'s in distances, angles and torsion angles; correlations between e.s.d.'s in cell parameters are only used when they are defined by crystal symmetry. An approximate (isotropic) treatment of cell e.s.d.'s is used for estimating e.s.d.'s involving l.s. planes.
Refinement. Refinement of *F*^2^ against ALL reflections. The weighted *R*-factor *wR* and goodness of fit *S* are based on *F*^2^, conventional *R*-factors *R* are based on *F*, with *F* set to zero for negative *F*^2^. The threshold expression of *F*^2^ > σ(*F*^2^) is used only for calculating *R*-factors(gt) *etc*. and is not relevant to the choice of reflections for refinement. *R*-factors based on *F*^2^ are statistically about twice as large as those based on *F*, and *R*- factors based on ALL data will be even larger.

### Fractional atomic coordinates and isotropic or equivalent isotropic displacement parameters (Å^2^)

**Table:**

	*x*	*y*	*z*	*U*_iso_*/*U*_eq_	
S1	−0.27417 (18)	0.2513 (3)	0.65551 (8)	0.0556 (4)	
Cl	0.2514 (3)	0.2819 (5)	0.47181 (11)	0.1051 (8)	
N1	−0.0494 (5)	0.1770 (7)	0.7725 (2)	0.0353 (10)	
O1	−0.0704 (5)	−0.2937 (7)	0.8136 (2)	0.0621 (12)	
C1	−0.1971 (6)	0.0662 (9)	0.7899 (3)	0.0379 (13)	
H1A	−0.2563	0.1677	0.8185	0.045*	
O2	−0.2241 (5)	−0.1623 (7)	0.8928 (2)	0.0531 (11)	
H2C	−0.1984	−0.2805	0.9148	0.080*	
C2	−0.3015 (7)	0.0154 (11)	0.7147 (3)	0.0519 (16)	
H2A	−0.4163	−0.0005	0.7195	0.062*	
H2B	−0.2653	−0.1222	0.6940	0.062*	
O3	0.0636 (4)	0.1875 (6)	0.89459 (18)	0.0422 (9)	
C3	−0.0548 (6)	0.2640 (10)	0.6970 (3)	0.0415 (12)	
H3A	−0.0212	0.4217	0.7007	0.050*	
O4	0.1931 (4)	0.3384 (6)	0.80485 (18)	0.0407 (9)	
C4	0.0541 (7)	0.1417 (9)	0.6516 (3)	0.0395 (13)	
C5	0.0950 (7)	0.2482 (12)	0.5896 (3)	0.0505 (14)	
H5C	0.0531	0.3897	0.5759	0.061*	
C6	0.1987 (8)	0.1427 (12)	0.5482 (3)	0.0592 (17)	
C7	0.2659 (8)	−0.0660 (13)	0.5677 (4)	0.0651 (19)	
H7A	0.3366	−0.1334	0.5394	0.078*	
C8	0.2260 (8)	−0.1720 (11)	0.6297 (3)	0.0590 (17)	
H8A	0.2689	−0.3131	0.6433	0.071*	
C9	0.1218 (7)	−0.0688 (10)	0.6721 (3)	0.0487 (15)	
H9A	0.0970	−0.1402	0.7144	0.058*	
C10	−0.1541 (6)	−0.1503 (10)	0.8333 (3)	0.0390 (13)	
C11	0.0700 (6)	0.2343 (9)	0.8296 (3)	0.0366 (11)	
C12	0.3466 (7)	0.4010 (9)	0.8560 (3)	0.0424 (14)	
C13	0.4314 (7)	0.1879 (12)	0.8896 (4)	0.0663 (19)	
H13A	0.3687	0.1236	0.9243	0.099*	
H13B	0.5400	0.2238	0.9152	0.099*	
H13C	0.4388	0.0823	0.8504	0.099*	
C14	0.4440 (8)	0.5092 (12)	0.8017 (4)	0.0635 (19)	
H14A	0.3894	0.6438	0.7819	0.095*	
H14B	0.4516	0.4071	0.7615	0.095*	
H14C	0.5525	0.5451	0.8274	0.095*	
C15	0.3093 (9)	0.5670 (12)	0.9141 (4)	0.073 (2)	
H15A	0.2574	0.6973	0.8894	0.110*	
H15B	0.4099	0.6100	0.9457	0.110*	
H15C	0.2369	0.4989	0.9438	0.110*	
O5	0.8493 (5)	0.4638 (7)	0.9669 (2)	0.0585 (12)	
H5B	0.7943	0.4847	1.0019	0.070*	
H5A	0.9053	0.3446	0.9659	0.070*	

### Atomic displacement parameters (Å^2^)

**Table:**

	*U*^11^	*U*^22^	*U*^33^	*U*^12^	*U*^13^	*U*^23^
S1	0.0481 (8)	0.0689 (11)	0.0474 (8)	0.0106 (9)	0.0013 (6)	0.0065 (8)
Cl	0.1239 (18)	0.130 (2)	0.0752 (12)	0.0239 (16)	0.0570 (12)	0.0417 (14)
N1	0.041 (2)	0.034 (2)	0.030 (2)	−0.003 (2)	0.0042 (19)	−0.0031 (19)
O1	0.076 (3)	0.049 (3)	0.069 (3)	0.013 (2)	0.031 (2)	0.005 (2)
C1	0.037 (3)	0.038 (3)	0.040 (3)	−0.002 (2)	0.011 (2)	−0.004 (2)
O2	0.061 (3)	0.048 (3)	0.055 (2)	0.005 (2)	0.021 (2)	0.012 (2)
C2	0.042 (3)	0.061 (4)	0.052 (4)	−0.007 (3)	0.005 (3)	−0.006 (3)
O3	0.047 (2)	0.046 (2)	0.034 (2)	−0.0027 (18)	0.0101 (16)	0.0039 (17)
C3	0.046 (3)	0.035 (3)	0.041 (3)	−0.001 (3)	0.001 (2)	−0.001 (3)
O4	0.042 (2)	0.041 (2)	0.0399 (19)	−0.0063 (18)	0.0093 (16)	−0.0023 (18)
C4	0.042 (3)	0.040 (3)	0.035 (3)	−0.001 (3)	0.002 (2)	−0.002 (3)
C5	0.061 (4)	0.046 (3)	0.047 (3)	0.001 (3)	0.017 (3)	0.003 (3)
C6	0.064 (4)	0.070 (5)	0.045 (4)	0.000 (4)	0.014 (3)	0.006 (3)
C7	0.063 (4)	0.082 (5)	0.054 (4)	0.011 (4)	0.023 (3)	−0.008 (4)
C8	0.077 (4)	0.049 (4)	0.055 (4)	0.015 (3)	0.023 (3)	0.000 (3)
C9	0.059 (4)	0.051 (4)	0.039 (3)	0.003 (3)	0.016 (3)	0.006 (3)
C10	0.035 (3)	0.041 (3)	0.042 (3)	−0.003 (3)	0.006 (2)	−0.002 (3)
C11	0.044 (3)	0.027 (3)	0.041 (3)	0.006 (3)	0.012 (2)	0.000 (2)
C12	0.037 (3)	0.039 (3)	0.051 (3)	−0.010 (3)	0.004 (3)	−0.001 (3)
C13	0.044 (3)	0.066 (5)	0.086 (5)	0.003 (3)	0.004 (3)	0.023 (4)
C14	0.052 (4)	0.059 (4)	0.080 (5)	−0.014 (3)	0.009 (4)	0.017 (4)
C15	0.079 (5)	0.067 (5)	0.071 (5)	−0.021 (4)	0.004 (4)	−0.021 (4)
O5	0.079 (3)	0.047 (3)	0.054 (3)	0.008 (2)	0.024 (2)	0.006 (2)

### Geometric parameters (Å, °)

**Table:**

S1—C2	1.807 (6)	C5—H5C	0.9300
S1—C3	1.838 (5)	C6—C7	1.384 (10)
Cl—C6	1.733 (6)	C7—C8	1.379 (9)
N1—C11	1.346 (6)	C7—H7A	0.9300
N1—C3	1.457 (6)	C8—C9	1.390 (8)
N1—C1	1.466 (6)	C8—H8A	0.9300
O1—C10	1.192 (6)	C9—H9A	0.9300
C1—C2	1.513 (7)	C12—C15	1.515 (8)
C1—C10	1.524 (7)	C12—C14	1.516 (8)
C1—H1A	0.9800	C12—C13	1.527 (8)
O2—C10	1.309 (6)	C13—H13A	0.9600
O2—H2C	0.8200	C13—H13B	0.9600
C2—H2A	0.9700	C13—H13C	0.9600
C2—H2B	0.9700	C14—H14A	0.9600
O3—C11	1.221 (6)	C14—H14B	0.9600
C3—C4	1.505 (7)	C14—H14C	0.9600
C3—H3A	0.9800	C15—H15A	0.9600
O4—C11	1.332 (6)	C15—H15B	0.9600
O4—C12	1.483 (6)	C15—H15C	0.9600
C4—C5	1.383 (8)	O5—H5B	0.8499
C4—C9	1.398 (8)	O5—H5A	0.8500
C5—C6	1.382 (8)		
			
C2—S1—C3	90.2 (3)	C7—C8—H8A	119.9
C11—N1—C3	122.2 (4)	C9—C8—H8A	119.9
C11—N1—C1	118.3 (4)	C8—C9—C4	120.5 (5)
C3—N1—C1	118.0 (4)	C8—C9—H9A	119.7
N1—C1—C2	105.2 (4)	C4—C9—H9A	119.7
N1—C1—C10	111.4 (4)	O1—C10—O2	124.6 (5)
C2—C1—C10	110.0 (5)	O1—C10—C1	123.3 (5)
N1—C1—H1A	110.0	O2—C10—C1	112.1 (5)
C2—C1—H1A	110.0	O3—C11—O4	126.3 (5)
C10—C1—H1A	110.0	O3—C11—N1	122.6 (5)
C10—O2—H2C	109.5	O4—C11—N1	111.1 (4)
C1—C2—S1	105.6 (4)	O4—C12—C15	110.3 (5)
C1—C2—H2A	110.6	O4—C12—C14	101.0 (4)
S1—C2—H2A	110.6	C15—C12—C14	111.5 (5)
C1—C2—H2B	110.6	O4—C12—C13	108.9 (4)
S1—C2—H2B	110.6	C15—C12—C13	113.6 (5)
H2A—C2—H2B	108.7	C14—C12—C13	110.9 (5)
N1—C3—C4	114.5 (4)	C12—C13—H13A	109.5
N1—C3—S1	103.9 (3)	C12—C13—H13B	109.5
C4—C3—S1	113.2 (4)	H13A—C13—H13B	109.5
N1—C3—H3A	108.3	C12—C13—H13C	109.5
C4—C3—H3A	108.3	H13A—C13—H13C	109.5
S1—C3—H3A	108.3	H13B—C13—H13C	109.5
C11—O4—C12	121.7 (4)	C12—C14—H14A	109.5
C5—C4—C9	119.1 (5)	C12—C14—H14B	109.5
C5—C4—C3	118.2 (5)	H14A—C14—H14B	109.5
C9—C4—C3	122.6 (5)	C12—C14—H14C	109.5
C6—C5—C4	119.5 (6)	H14A—C14—H14C	109.5
C6—C5—H5C	120.3	H14B—C14—H14C	109.5
C4—C5—H5C	120.3	C12—C15—H15A	109.5
C5—C6—C7	121.9 (6)	C12—C15—H15B	109.5
C5—C6—Cl	118.6 (5)	H15A—C15—H15B	109.5
C7—C6—Cl	119.4 (5)	C12—C15—H15C	109.5
C8—C7—C6	118.6 (6)	H15A—C15—H15C	109.5
C8—C7—H7A	120.7	H15B—C15—H15C	109.5
C6—C7—H7A	120.7	H5B—O5—H5A	120.0
C7—C8—C9	120.3 (6)		
			
C11—N1—C1—C2	−177.5 (5)	C4—C5—C6—Cl	−178.7 (5)
C3—N1—C1—C2	16.0 (6)	C5—C6—C7—C8	0.9 (11)
C11—N1—C1—C10	−58.3 (6)	Cl—C6—C7—C8	178.4 (5)
C3—N1—C1—C10	135.3 (5)	C6—C7—C8—C9	−0.8 (10)
N1—C1—C2—S1	−37.1 (5)	C7—C8—C9—C4	1.1 (10)
C10—C1—C2—S1	−157.2 (4)	C5—C4—C9—C8	−1.5 (9)
C3—S1—C2—C1	39.2 (4)	C3—C4—C9—C8	−177.9 (5)
C11—N1—C3—C4	82.2 (6)	N1—C1—C10—O1	−51.4 (7)
C1—N1—C3—C4	−111.8 (5)	C2—C1—C10—O1	64.9 (7)
C11—N1—C3—S1	−153.8 (4)	N1—C1—C10—O2	130.8 (5)
C1—N1—C3—S1	12.1 (5)	C2—C1—C10—O2	−112.9 (5)
C2—S1—C3—N1	−29.0 (4)	C12—O4—C11—O3	3.2 (8)
C2—S1—C3—C4	95.7 (4)	C12—O4—C11—N1	−174.5 (4)
N1—C3—C4—C5	−161.1 (5)	C3—N1—C11—O3	169.7 (5)
S1—C3—C4—C5	80.0 (6)	C1—N1—C11—O3	3.9 (7)
N1—C3—C4—C9	15.4 (8)	C3—N1—C11—O4	−12.4 (6)
S1—C3—C4—C9	−103.5 (6)	C1—N1—C11—O4	−178.3 (4)
C9—C4—C5—C6	1.5 (9)	C11—O4—C12—C15	−62.5 (6)
C3—C4—C5—C6	178.1 (5)	C11—O4—C12—C14	179.5 (5)
C4—C5—C6—C7	−1.2 (10)	C11—O4—C12—C13	62.7 (6)

### Hydrogen-bond geometry (Å, °)

**Table:**

*D*—H···*A*	*D*—H	H···*A*	*D*···*A*	*D*—H···*A*
O2—H2C···O5^i^	0.82	1.80	2.620 (6)	177
O5—H5A···O3^ii^	0.85	2.20	2.890 (5)	139
O5—H5B···O3^iii^	0.85	2.37	2.827 (5)	114

Symmetry codes: (i) *x*−1, *y*−1, *z*; (ii) *x*+1, *y*, *z*; (iii) −*x*+1, *y*+1/2, −*z*+2.
